# Automated Nonlinear Acoustics System for Real-Time Monitoring of Cement-Based Composites

**DOI:** 10.3390/s25216655

**Published:** 2025-10-31

**Authors:** Theodoti Z. Kordatou, Dimitrios A. Exarchos, Theodore E. Matikas

**Affiliations:** Mechanics, Smart Sensors & Nondestructive Evaluation (MSS-NDE) Laboratory, Department of Materials Science and Engineering, University of Ioannina, GR-45110 Ioannina, Greece; t.kordatou@uoi.gr (T.Z.K.); matikas@otenet.gr (T.E.M.)

**Keywords:** nonlinear acoustics, Laser Doppler Vibrometry, bulk waves, automated monitoring system, real-time material evaluation, cement-based composites, structural health monitoring, nondestructive evaluation, smart infrastructure, predictive maintenance

## Abstract

**Highlights:**

**What are the main findings?**

- Developed a fully automated nonlinear acoustics monitoring system combining bulk-wave excitation with non-contact Laser Doppler Vibrometry (LDV) detection.
- Validated the system’s stability, linearity, and repeatability, and demonstrated its capability for real-time monitoring of cement-based composites during hydration.

**What is the implication of the main finding?**

- Enables autonomous, high-sensitivity, and non-contact evaluation of material microstructure for smart infrastructure and predictive maintenance.
- Establishes a scalable framework for integrating nonlinear acoustic monitoring into digital twin and real-time structural health monitoring environments.

**Abstract:**

The development of automated systems for real-time material evaluation is becoming increasingly critical for structural engineering applications, infrastructure diagnostics and advanced material research. This work introduces a novel, fully automated nonlinear acoustics monitoring platform that employs Bulk Wave excitation in combination with non-contact Laser Doppler Vibrometry (LDV) detection to continuously assess the microstructural evolution of cement-based composites. Unlike conventional approaches—such as ultrasonic velocity measurements or compressive strength tests—which lack sensitivity to early-stage changes and also require manual operation, the proposed system enables unsupervised, high-precision monitoring of the material by leveraging the second and third harmonic generation (β_2_, β_3_) as nonlinear indicators of internal material changes. A specialized LabVIEW-based software manages excitation control, signal acquisition, frequency-domain analysis, and real-time feedback. As an initial step, the system’s stability, linearity, and measurement reliability were validated on metallic samples, and verified through long-duration experiments. Subsequently, the system was used to monitor hydration in cement-based specimens with varying water-to-cement and carbon nanotube (CNT) reinforcement ratios, thereby demonstrating its capability to resolve subtle nonlinear responses. The results highlight the system’s enhanced sensitivity, repeatability, and scalability, demonstrating that it as a powerful tool for structural health monitoring, smart infrastructure, and predictive maintenance applications.

## 1. Introduction

Cementitious materials remain indispensable in civil and structural engineering due to their mechanical strength, availability, and cost-efficiency [1,2,3]. The early-age evolution of their microstructure, governed by the hydration process, is critical in defining long-term durability and performance. Yet, despite their significance, most traditional assessment methods—including ultrasonic velocity measurements and compressive strength testing—lack the sensitivity required to detect early-stage microstructural changes [4,5]. These methods are also time-consuming, operator-dependent, and generally unsuitable for continuous or autonomous real-time health monitoring, as required by modern, data-driven infrastructure systems [6,7,8,9,10].

Over the past two decades, advances in nondestructive evaluation (NDE) have introduced powerful alternatives for structural material assessment [11,12]. While linear techniques such as acoustic emission and conventional ultrasonic testing have gained wide adoption [13,14,15,16], they often fall short in capturing the subtle nonlinear responses associated with early damage initiation or hydration dynamics [16,17,18]. In response, nonlinear acoustic methods—including second harmonic generation (SHG), nonlinear resonance, and wave modulation spectroscopy—have emerged as more sensitive tools for revealing hidden microstructural phenomena such as microcracking, interfacial debonding, or dislocation activity [4,19,20,21,22].

The early detection and monitoring of damage in engineering materials and structures remain a critical challenge for ensuring safety, durability, and cost-effective maintenance. Nonlinear ultrasonics has emerged as a highly sensitive technique for characterizing microstructural changes, fatigue-induced damage, and microcrack initiation at stages often undetectable by conventional linear ultrasonic methods. For metallic systems, nonlinear Rayleigh surface wave measurements have been shown to successfully identify fatigue-related microcrack growth in steels [23], while recent advances in nonlinear frequency mixing approaches have enabled non-contact detection of fatigue cracks through Rayleigh wave interactions [24]. Such approaches demonstrate the ability of nonlinear surface wave analysis to capture the earliest manifestations of fatigue, prior to macroscopic crack development.

In cementitious and concrete materials, the complexity of heterogeneous microstructures necessitates advanced sensing and analysis strategies. Recent experimental work has demonstrated the use of nonlinear ultrasonic parameters to track crack propagation in concrete under mechanical loading [25,26]. Building upon this, deep learning–assisted nonlinear ultrasonic analysis has been proposed as a powerful tool for identifying microcrack development with higher sensitivity and improved interpretability, highlighting the growing integration of artificial intelligence into nonlinear acoustic diagnostics [27]. Moreover, studies have confirmed that nonlinear ultrasonic techniques can characterize changes in cementitious materials during hydration and crack initiation using non-contact configurations such as air-coupled transducers [28].

The development of non-contact sensing technologies has further expanded the applicability of nonlinear ultrasonics in structural health monitoring (SHM). Laser Doppler Vibrometry (LDV), in particular, has shown significant promise as a tool for tracking nonlinear acoustic behavior in cementitious systems during the hardening process. LDV has also been deployed in large-scale infrastructures, such as hydropower dams, to capture changes in dynamic response under non-stationary conditions [29]. More recently, remote LDV sensing has been successfully implemented for the detection of subsurface cavities in tunnel linings, underscoring its potential for in situ monitoring of hard-to-access structures [30].

In addition to traditional acoustic and optical measurement strategies, machine learning techniques are increasingly leveraged to process nonlinear ultrasonic signals and correlate them with microstructural features. Applications include the prediction of crack density in rock materials based on ultrasonic data [31], as well as the classification of nonlinear signatures associated with fatigue damage in high-strength steels [32]. These developments highlight the dual trend of (i) integrating nonlinear ultrasonics with non-contact and remote sensing platforms, and (ii) fusing experimental measurements with advanced machine learning frameworks for enhanced feature extraction and damage characterization.

Taken together, these advances illustrate a rapidly evolving field where nonlinear ultrasonics, non-contact sensing, and artificial intelligence converge to enable highly sensitive, robust, and scalable approaches for monitoring materials and infrastructures across diverse environments.

Despite the promising results, widespread adoption of nonlinear NDE techniques remains constrained by technical and practical limitations. Most methods still rely on contact-based sensors, manual calibration, and post-processed data, thereby making the method inapplicable to modern smart infrastructure applications or digital twin environments [33,34,35]. In addition, inconsistencies in experimental protocols compromise repeatability and system stability—factors critical for long-term monitoring and large-scale deployment.

To address these challenges, this study introduces a fully autonomous nonlinear acoustic monitoring system that integrates high-power bulk wave excitation with LDV sensing and adaptive signal processing algorithms—achieving contactless, real-time assessment with no operator intervention.

The proposed system performs real-time acquisition, harmonic analysis, and nonlinear parameter computation using an integrated processing engine based on Fast Fourier Transform (FFT), adaptive filtering, and wavelet-based denoising for robust time-frequency representation [36,37,38].

The system’s performance in terms of stability, linearity, and repeatability was validated through more than 20 h of continuous monitoring.

In addition to benchmark validation, the system was applied to monitor the hydration of cement-based composites with varying water-to-cement (w/c) ratios and carbon nanotube (CNT) reinforcement [39,40]. While the full interpretation of these hydration-related results falls outside the scope of this paper, their inclusion highlights the platform’s versatility and suitability for real-world applications.

This work contributes to the field of computational NDE by presenting a deployable, non-contact, and autonomous framework for nonlinear acoustic monitoring. Owing to its operational independence and compatibility with a wide range of engineering materials—including metals and fiber-reinforced composites [28,29]—and its scalability for integration with cloud-based data acquisition and analytics platforms [30,31], this technique demonstrates significant potential for enabling predictive maintenance frameworks, supporting digital twin implementations, and advancing real-time structural health monitoring in smart infrastructure systems [32,33,34,35].The rest of the paper is structured as follows:

- Section 2 outlines the system design and methodological framework.
- Section 3 presents experimental validation and results.
- Section 4 concludes with future research directions and potential applications.

## 2. Methodology

### 2.1. Theoretical Background and Nonlinear Acoustic Indicators

In nonlinear acoustics, microstructural changes within a material are revealed through the generation of higher-order harmonics during ultrasonic wave propagation. Cement-based composites, particularly during early hydration, exhibit an intrinsic nonlinearity that can be detected through this mechanism, making it ideal candidate for real-time structural evaluation.

To probe the material’s nonlinear characteristics, a narrowband ultrasonic toneburst was transmitted at a fundamental frequency f_1_. In a purely linear medium, the response would be expected to replicate the excitation, producing energy only at f_1_. However, nonlinearities within the material give rise to harmonic generation, resulting in additional frequency components at 2f_1_, 3f_1_, and beyond. These harmonics were captured in the received signal and analyzed in the frequency domain, offering insights into the underlying nonlinear mechanisms.

Two nonlinear indicators are used to quantify the degree of nonlinearity [4]:

(1) β2=A2A12

(2) β3=A3A13

where A_1_ A_2_ and A_3_ are the amplitudes of the fundamental, second, and third harmonics, respectively. β_2_ is typically more sensitive to early-stage hydration and microdefect nucleation, whereas β_3_ reflects later-stage degradation phenomena.

To ensure computational accuracy, Parseval’s theorem [41] was applied to verify energy conservation between time- and frequency-domain representations, minimizing spectral inconsistencies and validating the integrity of the extracted harmonic amplitudes.

To record these harmonics with high precision, a non-contact LDV was employed. Utilizing the Doppler effect, the LDV enables surface velocity measurements with directional sensitivity and high bandwidth, making it particularly effective for detecting subtle nonlinear distortions.

The LDV signals were digitized and transformed into the frequency domain using Fast Fourier Transform (FFT), often after applying a suitable window function to minimize spectral leakage. The amplitudes were extracted and used in real-time to compute the nonlinear parameters.

### 2.2. System Architecture and Measurement Framework

The automated monitoring system integrates synchronized hardware modules for ultrasonic excitation, non-contact sensing, and high-speed data acquisition. Narrowband ultrasonic toneburst pulses—consisting of 5 cycles at a central frequency of 200 kHz—were generated using a RITEC RPR−4000 high-power, linear pulser/receiver unit (RITEC, Rochester, NY, USA). These pulses were transmitted through a piezoelectric transducer (Olympus V153), which was mounted on the rear face of a cementitious mold using a torque-controlled fixture to ensure repeatable coupling and minimize variability.

Non-contact surface velocity measurements were carried out using a PSV−400 Laser Doppler Vibrometer (Polytec), aligned perpendicularly to the front surface of the specimen at a fixed distance of 50 mm from the excitation point. To ensure reliable optical feedback and signal stability, a small patch of reflective paper was affixed to the specimen surface at the measurement location. This geometry enabled precise, non-contact detection of wave propagation without interfering with the material during its early-age evolution.

The system’s digitization and control functions were handled via a National Instruments PXIe−1071 chassis, comprising:

— PXIe−5105: high-speed oscilloscope module (12-bit, 60 MS/s)
— PXIe−8430: serial communication interface to RITEC
— PXIe−8360: synchronization and remote host control module

**Figure 1:**
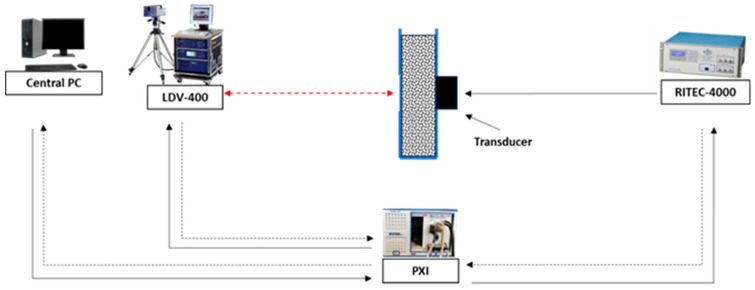
Schematic Diagram of the Automated Nonlinear Acoustic Monitoring System.

This robust experimental configuration ensured stable transmission paths and high-resolution surface response acquisition, enabling autonomous operation for both laboratory-scale evaluations and potential field deployments. A visual representation of the system architecture, detailing its components and workflow, is shown in Figure 1.

### 2.3. Signal Processing and Software Integration

**Figure 2:**
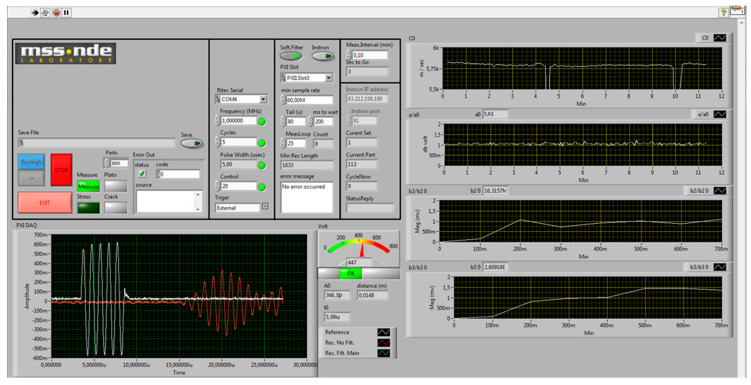
Real-time control and visualization graphical user interface (GUI) for nonlinear acoustic monitoring.

A dedicated LabVIEW-based software suite (Figure 2) was employed to orchestrate the monitoring system and to automate the entire workflow—from excitation parameterization (frequency, number of cycles, amplitude) and synchronized acquisition from the RITEC RPR−4000 pulser/receiver and the LDV, to real-time signal processing, visualization, and data archiving. Communication with the hardware was established through a National Instruments PXIe−1071 chassis comprising the PXIe−8360 for host control, the PXIe−8430/2 for serial communication with the RITEC unit, and the PXIe−5105 oscilloscope for high-speed digitization. Through this integration, centralized configuration of test conditions, live monitoring of responses, and systematic storage of results suitable for both laboratory and field operation were enabled.

**Hardware Initialization**—At startup, an automated hardware bring-up sequence was performed, during which the serial communication link to the RITEC unit was established and VISA resources were bound to fixed aliases to avoid port drift. Subsystems were locked to the PXI backplane time base, and the trigger topology was configured so that the RITEC and the digitizer acquisition share a common deterministic reference. Default acquisition parameters were applied to the PXIe−5105 with at least an order-of-magnitude oversampling relative to the 200 kHz toneburst (typically ≥2 MS/s), ensuring preservation of the transient rise and adequate anti-alias margins. Input ranges were negotiated to maximize the effective number of bits without risking clipping, and the LDV decoder bandwidth and velocity range were queried to maintain valid scaling to physical units. A short readiness routine verified LDV optical signal quality; runs were blocked if the photodetector signal-to-noise ratio (SNR) fell below threshold, a safeguard supported in practice by affixing a small reflective patch at the measurement spot to stabilize the return. This control ensured stable optical return and consistent fringe contrast throughout the measurements. Under these conditions, the LDV signal quality indicator remained close to 100%, corresponding to SNR values above 40 dB. A quantitative rejection threshold of 30 dB was empirically defined to guarantee reproducible harmonic amplitude extraction.

**Excitation Control**—The toneburst central frequency, number of cycles, and amplitude (and, where applicable, pulse width) were specified by the user, and the burst duration

(3) $$T_{b}\;=\;N_{cycles}\;/{\;f}_{c}$$

where T_b_ is the burst duration, N_cycles_ the number of cycles in the toneburst, and f_c_ the central (carrier) frequency, pre/post-trigger guard bands, and safe duty cycle were derived automatically. These parameters were committed to the RITEC with an explicit handshake that confirms acceptance and arms the unit for deterministic firing. Amplitude and repetition-rate limits were enforced to prevent transducer overheating or amplifier saturation, and the final device-acknowledged settings were logged together with the run metadata to ensure traceability.

**Waveform Acquisition**—Acquisition was synchronized by routing a hardware trigger from the excitation sequence to the PXIe−5105, ensuring a fixed phase relationship between the emitted burst and the recorded LDV response, thereby eliminating software-induced jitter. For each shot, the raw waveform was stored together with a detailed metadata bundle (timestamp, f_c_, number of cycles, drive amplitude, input ranges, filter and window selections, trigger times, and geometric offsets). Optional coherent or ensemble averaging (e.g., 4–64 repetitions) could be enabled to improve SNR in stable conditions; averaging was performed before normalization so that amplitude fidelity was preserved.

**Signal Conditioning and Segmentation**—The raw LDV voltage was first filtered using the digitizer’s anti-alias path, followed by a software band-pass centered on f_c_ with tunable bounds (typically ±10–20%) to suppress low-frequency drift and out-of-band noise. Where required, the signal was converted to physical surface velocity units via the LDV sensitivity S_v_ using

(4) $$v(t)\;=\;S_{v}\;*\;V_{LDV(t)}$$

where v(t) is the instantaneous surface velocity, S_v_ the LDV sensitivity coefficient, and V_LDV(t)_ the measured LDV voltage signal, with the selected decoder range recorded for reproducibility. The start of the informative portion was identified by a thresholding scheme: a primary threshold at 50% of the waveform peak detects onset, and the gate start was shifted earlier by ~20% of the local rise interval to avoid truncation under noisy conditions. A secondary, lower threshold (10%) refines endpoint detection in the presence of post-burst oscillations. To ensure that only the most informative segment was carried into spectral analysis, an energy-based refinement was applied using Parseval’s theorem; the cumulative energy curve was computed, and the processing window was trimmed at the 50% cumulative-energy point:

(5) $$\sum_{n\;-\;0}^{N\;-\;1}{\left|x[n]\right|}^{2\;}=\;\frac{1}{N}\sum_{k\;-\;0}^{N\;-\;1}{\left|X[k]\right|}^{2\;}$$

where x[n] was the discrete time-domain signal, X[k] its discrete Fourier transform (DFT), and N the number of samples. Finally, a ringing-correction stage applied the analytic signal envelope (Hilbert transform [42]) to identify and exclude late-time transducer ring-down that could bias spectral estimates.

**Spectral Analysis and Harmonic Extraction**—The segmented waveform was transformed via FFT to obtain the magnitude spectrum and identify the fundamental and higher-order components. Spectra were energy-normalized per Parseval consistency to stabilize comparisons across acquisitions and input-level changes. Harmonic amplitudes were extracted automatically by adaptive peak tracking in narrow windows around f_1_, 2f_1_, and 3f_1_, with local noise floors estimated from flanking bands to mitigate residual leakage. The fully automated routine minimizes operator influence, suppresses transient artifacts, and yields repeatable harmonic estimates suitable for long-duration fatigue monitoring.

**Bulk-Wave Velocity Estimation**—Arrival times were estimated using a dual-threshold logic that combines a 10% early pick with a 50% confirmation to mitigate noise sensitivity. The effective window was cross-checked against the ring-down-corrected envelope to prevent premature picks from late reflections. Where a reference drive or template is available, cross-correlation is employed to refine the time-of-flight estimate. The bulk-wave velocity was computed as

(6) v = L / ∆t

with L the calibrated propagation distance and Δt the inter-pick delay (or the delay relative to a hardware time-zero). Uncertainty was reported from pick-to-pick jitter across repeats together with the sampling quantization bound ±1/F_s_, where F_s_ is the sampling frequency of the digitizer.

**Attenuation**—Attenuation was quantified by the input–output amplitude ratio, with the runtime value α tracked against the initial reference α_0_ to capture temporal changes. The decibel formulation is:

(7) $$\alpha \;=\;-\;20\;*\;\log10\;(\;V_{out}\;/\;V_{in})$$

where α_0_ denotes the baseline attenuation reference used to monitor temporal variations in α.

Results were only accepted when both input and output levels remain at least 3 dB above the measured noise floor to avoid bias from stochastic fluctuations. When comparing paths of different lengths or geometries, an optional geometric-spreading correction (e.g., 1/r factors, where r denotes the propagation distance in the spreading model) could be applied so that material attenuation was isolated from geometric effects.

**Nonlinear Parameter Computation**—Nonlinear indicators were computed in real time from harmonic content. Using the window-corrected amplitudes A_1_, A_2_, A_3_, the parameters β_2_ and β_3_ were formed as proxies for quadratic and cubic nonlinearity, respectively. The first measurement in a series defines the baseline values $\beta_{2_{0}}$ and $\beta_{3_{0}}$; subsequent measurements were reported as normalized ratios β_2_/$\beta_{2_{0}}$ and β_3_/$\beta_{3_{0}}$ to enable robust temporal trending insensitive to absolute gain variations. Drive-level checks ensure that the excitation remains within the calibrated linear regime; deviations (e.g., anomalous scaling of A_2_ with input) were flagged and withheld from trend updates.

**Quality Control, Validation, and Robustness**—Each processed frame undergoes quality control (QC) tests assessing spectral consistency (energy fraction within a small bin neighborhood around the targets), harmonic ordering, and SNR in the analysis bands. Redundant shots within a burst were screened using median-absolute-deviation or interquartile-range criteria to remove outliers prior to averaging or trend updates. Optical health was monitored continuously via LDV diagnostics; if fringe visibility or photodetector level degrade—conditions typically prevented by the reflective patch—acquisition was paused and the operator was notified.

**GUI and Auxiliary Programs**—The graphical interface presents synchronized views of the time-domain waveform with gating overlays, the magnitude spectrum with harmonic markers and window annotations, and rolling trend plots for bulk-wave velocity, attenuation, harmonic amplitudes, and nonlinear ratios. A parameter console exposes excitation settings, filter and window choices, averaging depth, and QC thresholds; all changes are logged with timestamps. Three auxiliary LabVIEW routines run alongside the main loop: two provide high-rate visualization of raw and harmonic content for operator awareness, and one performs focused bulk-wave velocity estimation useful during calibration or geometry changes. Their outputs were fed back into the main process in real time.

**Figure 3:**
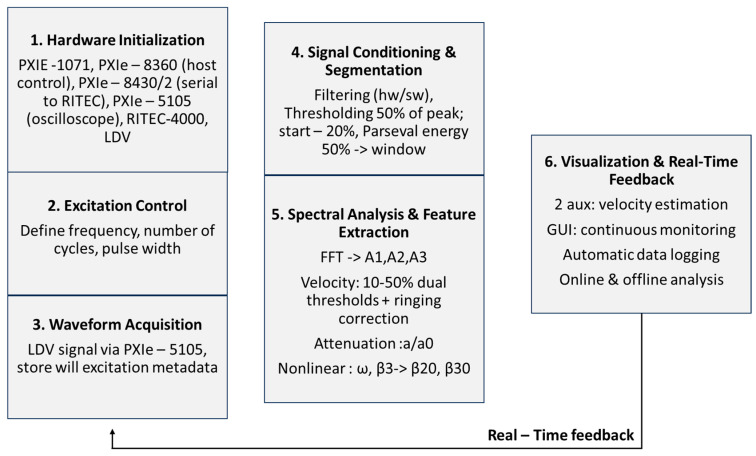
Schematic of the software workflow—from initialization and excitation control through acquisition, conditioning, spectral analysis, velocity and attenuation estimation, nonlinear metrics, and visualization—with a real-time feedback loop enabling fully.

The overall architecture employs producer–consumer loops with direct memory access (DMA)-backed queues to prevent acquisition stalls during processing or logging. Within each cycle, the trigger initiates acquisition, after which conditioning, spectral and parameter extraction, QC, and archival proceed deterministically before the next burst. Latency budgets ensure that single-shot processing completes within the inter-burst interval, enabling continuous unattended operation. All raw data and metadata were synchronously logged into Technical Data Management Streaming (TDMS) files to preserve temporal consistency, with periodic Comma-Separated Values (CSV) exports of processed indicators. File rotation and checksum validation safeguarded against corruption. By coupling acquisition, processing, and logging in a closed loop, the platform provides reliable online visualization and robust offline analysis, and was directly compatible with higher-level digital-twin, structural-health-monitoring, and predictive-maintenance frameworks (Figure 3).

## 3. Experimental Validation

### 3.1. System Stability and Linearity Assessment

Before application of the nonlinear acoustic monitoring system to cementitious materials, verification was performed to ensure that the instrumentation itself did not introduce measurable nonlinearity or drift. The objective was to confirm that any nonlinear responses observed during hydration originated from the material rather than from the excitation, sensing, or processing chain. For assessment of long-term stability, a reference steel specimen (thickness 14.8 mm) was monitored continuously for 20 h under laboratory conditions. Ultrasonic excitation was provided by a 5 MHz piezoelectric transmitter, while two receiving transducers (nominal center frequencies 5 MHz and 10 MHz) were mounted on the opposite face along a common path. Higher-frequency (5 MHz) tonebursts were selected for the metallic validation tests to ensure clear harmonic separation and negligible attenuation, whereas lower-frequency (200 kHz) excitations were employed in cementitious specimens to maintain adequate penetration and signal fidelity under strongly attenuative conditions. This setup also enabled simultaneous observation of the fundamental (5 MHz) and its second harmonic (10 MHz) with receivers optimized for each band. Coupling was controlled by means of a torque-limited fixture to mitigate drift, and ambient temperature was recorded adjacent to the specimen to track thermoelastic influences on wave speed.

**Figure 4:**
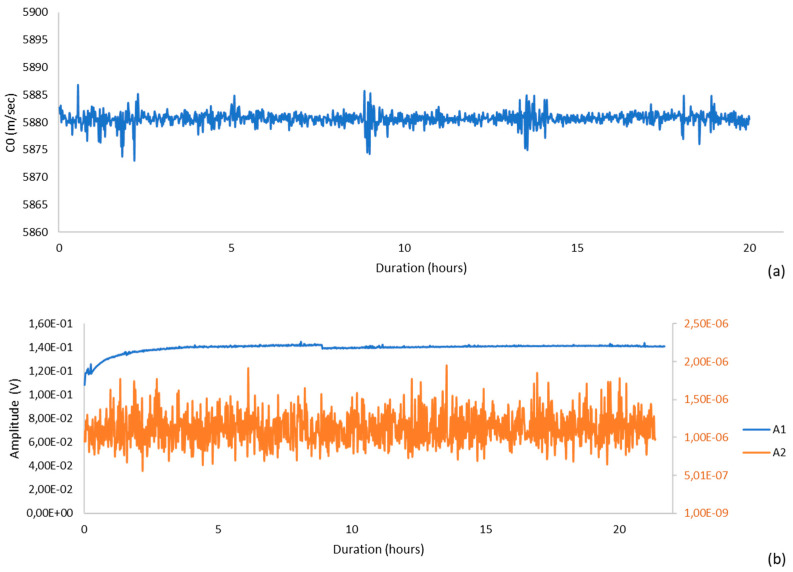
Stability Test at Room Temperature: (**a**) Wave Velocity, (**b**) Harmonic Amplitudes.

Time-of-flight and spectral amplitudes were processed using the same pipeline employed in the cementitious tests: gated tonebursts were windowed to minimize spectral leakage, FFT magnitudes were corrected for window coherent gain, and arrival times were picked with a dual-threshold method (10%/50%) including ring-down correction. Ultrasonic velocity remained effectively constant throughout the 20 h observation, exhibiting only minor fluctuations coincident with small ambient temperature variations (Figure 4a). After accounting for this weak temperature dependence, the residual velocity variations were consistent with the expected limits set by pick jitter and sampling quantization, and no monotonic trend indicative of instrumental drift was detected.

Spectral amplitudes demonstrated comparable stability. The fundamental amplitude recorded by the 5 MHz receiver exhibited small, non-systematic variations over time, whereas the 10 MHz channel—used to monitor the second harmonic—remained near the noise floor without systematic growth (Figure 4b). Expressed as normalized indicators, both the fundamental and second-harmonic traces remained within narrow bands over the full duration, with no evidence of progressive degradation in coupling, transducer performance, or digitizer gain.

To assess linearity explicitly, the system was exercised over a controlled range of input drive levels while duty cycle, gating parameters, and geometry were held fixed. The fundamental response scaled proportionally with input, and the second-harmonic response did not exhibit a correlated increase with drive, confirming that the combined electronics–transducer–processing chain did not generate measurable self-nonlinearity in the operating regime used here.

Taken together, these observations indicate that the system is stable on day-long time scales, repeatable on short time scales, and linear over the excitation range adopted for subsequent hydration experiments, thereby supporting attribution of nonlinear trends to the cement-based specimens.

**Figure 5:**
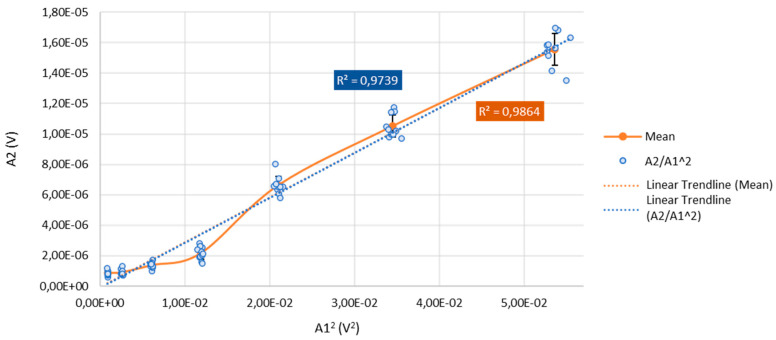
Linearity Assessment of the Measurement System via the Nonlinear Ratio A_2_/A_1_^2^.

To further characterize the instrumentation, a transmitter-voltage sweep on the steel reference was performed and the deterministic second-harmonic component generated by the measurement chain was quantified. The input voltage to the transmitter was stepped over a controlled range, and at each level ten repetitions were acquired under identical conditions. Individual measurements (blue points) together with the ten-shot average (orange curve) are shown in Figure 5. The second-harmonic amplitude (A_2_) increased monotonically with drive and exhibited a strong linear relationship with the squared fundamental (A_1_^2^), with correlation coefficients R2 = 0.9739 for the raw data and R2 = 0.9864 for the averaged series. This behavior is consistent with a weak, but repeatable, quadratic nonlinearity in the transducer–electronics chain.

The small error bars across the sweep indicated excellent repeatability, and the deterministic scaling enabled robust compensation of instrument-induced second-harmonic content when interpreting nonlinear responses in metallic specimens.

These results confirmed that the measurement system exhibited linear behavior throughout the tested range. Consequently, the nonlinearities observed in subsequent hydration experiments could be confidently attributed to the evolving material microstructure rather than instrumental or measurement artifacts.

### 3.2. Real-Time Nonlinear Acoustic Evaluation of Cement-Based Materials

Following verification of the system’s linearity and long-term stability (Section 3.1), we emphasize its applicability to the early hydration of cement-based composites under controlled curing conditions. As reported in our previous studies [26,27], cement paste specimens were prepared with various water-to-cement (w/c) ratios, both plain and reinforced with carbon nanotubes. For completeness, we note that the same automated LDV-ultrasound platform has also been applied in our previous work to fine-aggregate mortars, under comparable curing conditions and with the same nonlinear harmonic analysis workflow [26,27].

Real-time ultrasonic monitoring commenced immediately after casting and continued uninterrupted for 48 h. Excitation was provided by a 5-cycle toneburst centered at 200 kHz, generated via the RITEC RPR−4000 and applied through an Olympus V153 transducer. Surface velocity responses were captured by a Polytec PSV−400 Laser Doppler Vibrometer (LDV) positioned 50 mm from the excitation point. Signals were digitized with the PXIe−5105 oscilloscope at 12-bit resolution and 60 MS/s, oversampling by nearly two orders of magnitude relative to the central frequency to preserve transient fidelity. Harmonic amplitudes (A_1_, A_2_, A_3_) were extracted automatically using the LabVIEW interface, which performed gating, FFT analysis, and normalization. The nonlinear parameters β_2_ = A_2_/A_1_^2^ and β_3_ = A_3_/A_1_^3^ were computed continuously, with a resolution better than ±0.01.

As reported in [26,27], the system successfully captured formulation-dependent nonlinear signatures: CNT-reinforced pastes exhibited elevated β_2_ values within the first 24 h of hydration (≈20–30% higher than plain pastes), while higher w/c ratios showed a delayed onset in β_3_ evolution (~6 h relative to low w/c mixes). These distinct temporal trends confirmed the sensitivity of the nonlinear indices to subtle variations in microstructural development during hydration. Across the three replicate specimens (n = 3), the evolution of β_2_ and β_3_ exhibited consistent temporal trends, with variations remaining within the typical repeatability limits (<5%) [26,27].

A full mechanistic interpretation of these behaviors is beyond the scope of the present work. Instead, we point the reader to [26,27], where the same automated system developed in this study—including the hardware configuration, LDV-based sensing, data acquisition framework, and custom LabVIEW software—was applied systematically to cement pastes and mortars. Those studies demonstrated that the evolution of β_2_ and β_3_ correlates with hydration-induced phase transitions, progressive stiffening, and nonlinear scattering phenomena. In the context of the present paper, this body of prior work provides strong independent validation of the system’s precision and reproducibility, confirming its suitability for autonomous monitoring of cementitious materials.

## 4. Discussion

This study presents a novel, fully automated system for real-time nonlinear acoustics monitoring of cementitious materials that unites bulk wave excitation, non-contact Laser Doppler Vibrometry (LDV) detection, and advanced signal processing into a seamless, autonomous framework. By eliminating the need for manual calibration and operator intervention, the system ensures continuous, unsupervised operation—an essential step toward intelligent, data-driven infrastructure monitoring.

Validation experiments confirmed the system’s long-term stability, linearity, and repeatability, enabling reliable detection of nonlinear signatures during the early hydration of cement-based materials. The automated workflow—from excitation control and high-speed acquisition to spectral analysis, nonlinear indicator computation, and live feedback—demonstrates that nonlinear acoustics monitoring can be performed with efficiency and precision.

Regarding the applicability to more heterogeneous cement-based materials, the same measurement principle remains valid provided that the excitation frequency is appropriately selected with respect to aggregate size. Scattering effects become significant only when the characteristic aggregate dimension approaches the acoustic wavelength. At the 200 kHz operating frequency used here, the wavelength in cementitious media (~20 mm) is several times larger than typical fine-aggregate dimensions; under these sub-wavelength conditions, the medium behaves effectively homogeneous and supports stable nonlinear wave propagation with reproducible harmonic analysis. For coarse-aggregate concretes, the same concept can be extended by using lower excitation frequencies (longer wavelengths) to maintain the sub-wavelength condition and ensure accurate nonlinear parameter estimation under more heterogeneous conditions.

Beyond its immediate experimental application, the platform establishes a scalable foundation for integration into smart infrastructure and digital twin environments. Its autonomous operation, combined with real-time extraction of sensitive nonlinear parameters of second and third order (β_2_, β_3_), makes it particularly suited for predictive maintenance, large-scale structural health monitoring, and next-generation intelligent asset management systems. By bridging nonlinear acoustics diagnostics with full automation and real-time analytics, this work introduces a transformative tool that enables proactive decision-making and resilient infrastructure management.

## 5. Conclusions

In summary, this study presents a fully automated, real-time nonlinear acoustic monitoring platform that is sensitive, repeatable, and scalable. The system integrates bulk-wave excitation, non-contact LDV detection, and adaptive signal processing, enabling continuous and unsupervised evaluation of cement-based materials. Through systematic validation, it demonstrated long-term stability, linearity, and reproducibility, confirming both scientific rigor and practical relevance. As such, the platform constitutes a significant advancement in nonlinear nondestructive evaluation and provides a robust foundation for future intelligent monitoring solutions that support safer, smarter, and more sustainable infrastructures. Future work will extend the system to additional cement-based materials and curing regimes to further assess its robustness and long-term operational reliability.

## Data Availability

The raw data supporting the conclusions of this article will be made available by the authors on request.
